# Molecular Average and Intramolecular δ^13^C Measurements of Pyruvic Acid and Acetic Acid Using Gas Chromatography Orbitrap Mass Spectrometry (GC Orbitrap-MS)

**DOI:** 10.1021/acs.analchem.5c02194

**Published:** 2025-08-26

**Authors:** Youki J. Sato, Allison A. Baczynski, Hao Xie, Alexis Gilbert, Katherine H. Freeman

**Affiliations:** † Astrobiology Center for Isotopologue Research, Department of Geosciences, 311285 Pennsylvania State University, University Park, Pennsylvania 16802, United States; ‡ International Center for Isotope Effects Research, 12581 Nanjing University, Nanjing 210023, China; § Institute for Marine and Atmospheric Research Utrecht (IMAU), Utrecht University, Utrecht 3526 KV, the Netherlands

## Abstract

We developed a novel method using gas chromatography – Orbitrap mass spectrometry (GC Orbitrap MS) to measure the intramolecular carbon isotope value of pyruvic acid and acetic acid with high mass accuracy and mass resolution with nanomole (10–100) injections of analyte. Previous efforts to analyze intramolecular isotope patterns of small organic acids have been limited by labor-intensive chemical degradation steps, a narrow potential analyte pool, or large sample mass requirements. We present a new way to trap an analyte peak, which is then tested by measuring the molecular average and intramolecular carbon isotope values of pyruvic acid and acetic acid standards. Molecular average (MA) δ^13^C measurements with our method agreed with complementary methods within 2.2–2.6‰ relative mean standard deviation (RMSD) for 0.1 micromoles of compound, whereas intramolecular δ^13^C values varied from the complementary method from 1.9 to 4.9‰ RMSD. Intramolecular δ^13^C measurements of pyruvic acid and acetic acid yielded acquisition errors (σ_AE_) of better than 0.2‰ and an experimental reproducibility (σ_ER_) of <2.8‰ and <3.4‰, respectively, for 0.1 micromoles of analyte. This method enables measurements of the intramolecular δ^13^C values of small organic acids sufficiently precise to differentiate synthesis pathways for these compounds, including for pathways within the central metabolism, and in extracts of carbonaceous meteorites, which are theorized to have formed in interstellar ices.

## Graphical abstract

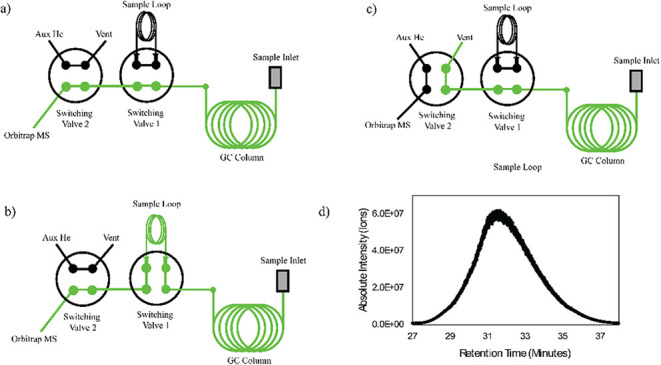

## Introduction

Studies have shown that the natural distribution of isotopomers for a given molecule is nonstochastic under nonequilibrium conditions, suggesting that different carbon sources and reaction processes may be reflected in each position on a molecule.^1^ As such, a molecule’s intramolecular isotopic patterns can be linked to specific steps involved in its synthesis.^1^

Past efforts to analyze the isotopic heterogeneity of organic molecules relied on labor-intensive chemical degradation steps,^2^ were limited to select highly volatile analytes, or used ^13^C nuclear magnetic resonance (NMR) spectroscopy, which requires hundreds of milligrams and long integration times.^3^ Isotope ratio-mass spectrometry (IRMS) systems coupled to pyrolysis-gas chromatography (py-GC IRMS) can measure position-specific isotope ratios of analytes such as acetic acid with high precision (±0.4 permil; ‰), but target analytes are typically limited to smaller compounds in order to assign carbon positions to the molecular gases formed during pyrolysis.^4^ Recently, high-resolution/high accuracy mass spectrometers (HR/HA MS) including Orbitrap MS systems used in fields such as metabolomics and proteomics are now being adapted for natural abundance isotope research.^5^

This work demonstrates the capabilities of a modified GC Orbitrap-MS system at Pennsylvania State University (Penn State) to measure carbon-isotope patterns within small organic acids. The Orbitrap’s high mass accuracy and mass resolution can differentiate isotopologues, enabling measurement of their ratios at natural isotope abundances.^6–8^ Direct measurements of analyte isotope ratios using a gas chromatography-electron impact (GC-EI) Orbitrap MS system is possible because of extended observation time using a bespoke sample introduction method.^9^ Using this method, molecules are separated via GC and introduced to the ion source for up to 5–8 min. Target fragment ions are selected via the Advanced Quadrupole Selector (AQS), passed through the automated gain control (AGC) gate, and collected in the C-trap until a user-defined threshold of ions is reached.

The packet of ions in the C-trap is released into the Orbitrap, where they oscillate at frequencies proportional to their mass that are converted to mass/charge values using fast Fourier transform methods.^5,10^ In this application, a user identifies isotopologue ratios (i.e., ^13^ *R* = ^13^C/^12^C or ^18^ *R* = ^18^O/^16^O) to quantify and use in position-specific isotope ratio calculations.^9^ A fragment ion must be attributed to a specific site in the parent molecular ion, which is done via the analysis of a standard isotopically labeled at specific positions. Intramolecular isotope ratios are calculated by solving the contribution of each carbon to the fragment’s isotope ratio.^11,12^

To increase the amount of time a target analyte is observed, a sample loop system was added to the GC flow path. For nanomole injections of tetradecane, the GC-sample loop-MS system at Penn State sustained high ion intensity for >10 min after it was released from the trap (Figure 1d; see Supporting Information for details regarding the preliminary experiments involving tetradecane). We adapted this method for analyses of pyruvic acid and acetic acid: two carboxylic acids essential for modern life,^13–15^ and that are also thought to be important reactants for prebiotic chemistry reactions on the path to the Origin of Life.^16–20^

**Figure 1:**
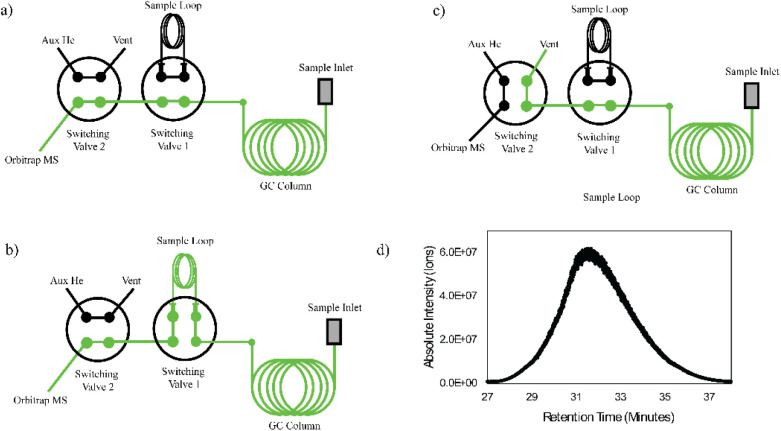
Schematic of the peak trapping system for the Orbitrap installed at Penn State for (a) direct injection or equilibration of analyte within the isolated sample loop, (b) sample loop elution, and (c) column maintenance. (d) Representative chromatogram of tetradecane after it has been trapped in a sample loop for 15 min, offering a high analyte signal over most of the elution period.

Intramolecular δ^13^C values of standards measured on the Orbitrap compared well with results via GC isotope ratio mass spectrometry (GC-IRMS)^21^ and GC-py-GC IRMS.^4^ For nanomole injections of each acid, molecular and intramolecular δ^13^C values agreed within 1.9–4.9‰ relative mean standard deviation (RMSD) and with experimental reproducibility (σ_ER_) of <2.8‰ and <4.9‰ for pyruvic acid and acetic acid, respectively.

## Materials and Methods

### GC-MS Conditions

Analyses were made using a Thermo Scientific (Thermo) Q-Exactive Orbitrap with mass resolutions up to 120,000 (fwhm), and a Supelco Nukol GC column (30 m × 0.25 mm × 0.25-μm phase thickness). The split/splitless inlet was set to a split configuration and kept at 240 °C, with a split flow of 40 mL/min introduced after an initial splitless period of 1 min. The helium flow was kept at 2 mL/min during peak separation and peak trapping. At 1 min before the trapping system was switched for MS analysis, carrier flow was lowered to 0.50 mL/min to maximize the width of the released peak. Programmed flows lower than 0.5 mL/min encountered flow fluctuations on our system causing errors (a limitation of the mass flow controller installed on our GC system combined with the increased volume from the sample loop), so 0.5 mL/min was chosen as the flow rate for peak release from the sample loop.

For the pyruvic acid, the GC oven temperature started at 60 °C, held for 1 min, then increased to 190 °C at 15 °C/min. This oven temperature was held at 190 °C to ensure the acid stayed in the gas phase and maximize its expansion within the sample loop, for a total run length of 55 min. For acetic acid, the GC oven started at 60 °C, held for 1 min, then heated to 140 °C at a rate of 15 °C/min and then held at this temperature 35.40 min. After MS analysis, the column temperature was increased to 190 °C at a rate of 30 °C/min to clear the column for the next analysis for a total run length of 50 min. The ion source and transfer line to the mass spectrometer were kept constant at 280 and 250 °C, respectively.

The operating parameters for the Orbitrap MS system were as follows: 1 microscan per cycle, an AGC target of 5 × 10^5^ ions, a maximum injection time of 120 ms, and a mass resolution of 60,000. Data was collected for the entirety of each acquisition; around 60 min. The mass window was set to *m*/*z* 40–49 to capture the 43 and 45 fragment ions and associated isotopologues, and was chosen to reduce space charge effects and the number of background or fragment ions unrelated to the target analyte that could interfere with accurate isotope ratio measurements.^22^

### Peak Trapping

A 10 mL stainless steel metal sample loop (SilcoTek; SL10KCSTP), treated with SilcoNert 2000 (SilcoTek) to prevent chemisorption and/or sample degradation, was installed between two four-port switching valves (VICI-Valco Instruments Co. Inc.). The valves are actuated by pneumatic solenoids (Humphrey; 310-24-VDC), to enable the different carrier gas flow configurations (Figure 1a–c). The solenoids were controlled by the “external events” function of the Aux Temperature/Cryo Module of the Trace 1310 GC.

The precision of isotope ratio measurements on the GC-Orbitrap MS system relies on the quantity of measured ions, particularly of rare isotopes. The number of observed ions is determined by how many packets of ions are observed, and how many “scans” of each mass fragment are collected for each packet. A scan is the span of time (milliseconds) in which the Orbitrap observes a packet of ions. Increasing the number of scans of a particular ion fragment improves the overall signal-to-noise ratios for each peak.^6^

Increasing the number of ions observed in total can be achieved by extending the observation time. We achieved this by modifying how the sample is introduced to the mass spectrometer via “peak capture.” The slow release of a captured peak to the ion source increases the amount of time a target analyte is observed, which can improve precision of isotope ratios down to tenths of a permil.^6,9^

Direct measurement of untrapped peaks enabled us to optimize chromatography and to confirm peak retention times. Untrapped peaks can also be used to determine precise isotope ratios for molecules with high ionization efficiency, such as polycyclic aromatic hydrocarbons (PAHs).^9^ However, isotope measurement via direct elution is not practical for molecules that do not ionize efficiently because ion counts are limited per scan.

The accuracy of the measured isotope ratios on the Orbitrap compared with other independent measurements of the ratios can reach 1‰ or better.^6,23^ This requires the number of ions observed per scan meets the AGC target (which is approximately the product of the total ion current (TIC) and injection time (IT, in ms). Under these conditions, the number of ions per scan is relatively consistent and stable. However, AGC target and mass resolution must be tuned to collect enough of the rare isotopologue, and as discussed later, the threshold of precision is determined by the shot noise limit (SNL) for a given ion fragment.

### Analysis of Samples

Pyruvic acid standards were purchased from various companies: Thermo Scientific (A0440271; Ther-B), Tokyo Chemical Industries (Lot: Z7WLE-RO), Sigma-Aldrich (Lot: SHBL6258 and STBL0221 for Sigma-A and Sigma-B, respectively), and Acros Organics (Lot: A0425850) (hereafter Thermo, TCI, Sigma, and Acros). Position-specific isotope ratios of samples are first reported relative to the TCI pyruvic acid, which served as a working standard, prior to converting them to the Vienna Pee Dee Belemnite (VPDB) scale. Pyruvate ^13^C-labeled at either the carboxyl or methyl carbon position were purchased from Sigma-Aldrich (99%; Lot: MBBD0927 and MBBC9019, respectively) to confirm the mass fragmentation pattern on the Orbitrap. The concentrated standards were diluted in water to mM levels so that ∼100 nanomoles of analyte would be introduced for a 1 μL injection. To prevent possible dimerization reactions between pyruvic acid monomers within the aqueous sample and improve chromatographic resolution,^24^ all samples (in ∼1 mL water) were acidified with 1 N HCl or via addition of 0.33 mmol oxalic acid to a pH < 2 prior to analysis.^25^

To complement the GC-Orbitrap measurements, all pyruvic acid lab standards were measured for their intramolecular δ^13^C values via the GC pyrolysis-GC IRMS (GC py-GC IRMS) system at the Tokyo Institute of Technology (Tokyo Tech; Table S2). Additionally, following methods adapted from Yamamoto & Back,^26^ Sigma-A was subjected to closed-tube pyrolysis and subsequent analysis via GC-IRMS to measure its carboxyl position liberated from pyruvic acid as CO_2_ (see method detailed in Supporting Information). The molecular average δ^13^C values of each standard was also measured via conventional GC-IRMS.

The intramolecular isotope ratios of the TCI and Sigma-A standards were measured via GC pyrolysis-GC IRMS. This enabled TCI to be used as the conversion standard for reporting isotope values measured on the GC Orbitrap on the Vienna Pee Dee Belemnite scale (δ^13^C_VPDB_). ^13^C/^12^C ratios (^13^ *R*) are reported using delta notation, expressed in permil (‰):

1 δCsample,VPDB13(‰)=(Rsample,VPDB13RVPDB13)−1

The methods for GC py-GC IRMS analysis at Tokyo Tech are also given in the Supporting Information.

Synthetic acetic acid stocks were purchased from Sigma (Lot: MKCQ4136), Acros (Lot: A0296026), and Millipore (Lot: AX0073-9). A volatile free acid mix of 10 different carboxylic acids, CRM46975 (Lot: LRAD2549), was purchased from Sigma and its acetic acid peak used as an additional sample for isotope analysis. Acetic acid ^13^C-labeled at the methyl carbon (99%; Lot: MBBC7055; Sigma) was used to confirm mass fragmentation patterns. Three isotopically characterized acetic acid standards from Tokyo Tech (standards AAA, AAB, and AAD; see Yamada et al.^27^ were measured using the GC-Orbitrap as a test of measurement accuracy.

The intramolecular isotope ratios of the standard, AAA, as measured for off-line combustion and pyrolysis products in sealed tubes by GC-C-IRMS^27^ provided conversion standard for reporting values on the VPDB scale.

Prior to all GC analyses of acetic acid samples, solutions were acidified with 1 N HCl or via addition of 0.33 mmol oxalic acid to a pH < 2 to improve chromatographic resolution.

The molecular average δ^13^C values of both pyruvic acid and acetic acid standards were measured via 252 GC-IRMS by adapting the methods described in Thomas et al.^25^ Specific run conditions for the GC-IRMS analyses are detailed in the Supporting Information.

### Orbitrap Data Processing

Sample analyses were paired with standards of known isotopic composition, under identical run conditions and approximate concentrations that were run directly after the sample. Instrument RAW files generated by the Orbitrap software (Xcalibur; Thermo) are converted using the FTStatistic software (Thermo) to extract ion intensities and other precision data per scan into a .txt file. Ion counts are calculated from the signal intensities (NL scores) reported in the RAW files following the conversion described by Eiler et al. and Zeichner et al.:^6,9^

2 $$N_{IO}=\left(\frac{S}{N}\right)\times \left(\frac{C_{N}}{z}\right)\times \sqrt{\frac{R_{N}}{R}}\times \sqrt{\mu }$$

*N*_IO_ is the approximate number of ions observed in the trap for a single cycle of measurement, *S* is the reported signal intensity (NL score; unitless) for the mass corresponding to the target fragment ion, *N* is the noise associated with that signal, measured at resolution *R* (defined as *m*/Δ*m*, where *m* is the mass of the target ion and Δ*m* is the difference in mass required to separate that ion from the nearest isobar); *C_N_* is an experimental constant (4.4) of the Orbitrap that quantifies the number of charges corresponding to the Orbitrap noise value at the resolution at which eq 2 was calculated, *R_N_*, μ is the number of microscans, and *z* is the charge of the ion at the mass of interest.

The .txt file is then processed using Python code written by the Eiler Lab at Caltech, which can be found in the Caltech GitHub data repository.^28^ The code allows the user to specify mass fragments of interest, the time frame in which to integrate peak areas, background noise subtractions, and a threshold to cull scans below the AGC target by including scans with greater than or equal to 20% of the maximum TIC count during peak elution. An intensity and ^13^ *R* profile for a representative acquisition of the 44 *m*/*z* fragment of acetic acid is shown in Figure 3a. The culling threshold of 20% (red bars) isolates the peak region (blue points) in which the ^13^ *R* value (yellow points) is most stable, which reduces the relative standard error for an acquisition. The TIC*IT profile of the same trapped peak (Figure 3b, green points) illustrates it achieves the specified AGC target of 5 × 10^5^ (dashed orange line) throughout the elution period.

^13^ *R* values are calculated as the ratio of ion counts of all singly substituted ^13^C isotopologues for a given fragment vs the unsubstituted ^12^C isotopologue. eq 3 shows the ^13^ *R* of the 43 fragment isotopologue (^13^ *R*_43_) for pyruvic acid, where Σ*i*_*x*_ is the sum of all isotopologues of mass *x*:

3 R4313=Σi44i43

The average ^13^ *R* across all scans is then reported, along with the relative standard error of this ratio across all scans. The relative standard error for the ratio (reported as the acquisition error once propagated across all calculations; σ_AE_) is calculated by dividing the standard deviation (SD; eq 4) between

individual scans of ^13^ *R* relative to the average ^13^ *R* value by the root of the total number of scans (eq 5):

4 SD=∑(Ri13−Ri13®)2n−1

5 $$\sigma_{AE}=\frac{\mathrm{SD}}{\sqrt{n}}$$

^13^ *R*_i_ denotes a single ^13^ *R* measurement, R13® is the average ^13^ *R* value for a given acquisition, and *n* is the number of scans in the acquisition (typically in the thousands). Each sample-standard pair was analyzed at least in triplicate and the average δ^13^C value of these acquisitions is reported. Experimental reproducibility errors (σ_ER_, in ‰) are first calculated for replicate acquisitions of the same sample, then are reported as the standard error between the calculated δ^13^C values for replicate standard-sample acquisitions (see Supporting Information).

Isotope abundances of fragments for pyruvic acid are initially calculated relative to the TCI standard (*R*_sam_/*R*_TCI_). Similarly, sample isotope ratios for acetic acid were calculated relative to the AAA standard prior to conversion to the VPDB scale (*R*_sam_/*R*_AAA_). eq 6) shows how the ^13^ *R* of a pyruvic acid sample would be reported using delta (δ) notation relative to the TCI standard ^13^ *R*:

6 δC43,TCI13(‰)=(Rsample,4313RTCI,4313)−1

Converting isotope ratios measured on the Orbitrap to the VPDB scale is possible in cases where the standard has been measured using a complementary measurement approach (^13^ *R*_standard,VPDB_).^7^ The sample isotope ratio can then be reported in δ notation relative to VPDB:

7 Rsample,VPDB13=(Rsample,Orbitrap13Rstandard,Orbitrap13)×Rstandard,VPDB13

In this study, the isotope abundances of pyruvic acid standard (TCI) was measured via GC-py-GC IRMS^4^ and the acetic acid standard (AAA) was measured via the methods described in Yamada et al.^27^ TCI and AAA were converted to the VPDB scale using the value of the ^13^ *R*_VPDB_ of pyruvic acid standard Sigma-A and acetic acid standard AAB.

### Definition of Shot Noise Limits

The shot noise limit (SNL) of the instrument is the theoretical best precision achievable if the only source of error is due to the stochastic nature of the ion current and the number of ions that reach the C-trap.^9,29^ SNLs are estimates based on the total number of ions detected (eq 2).^6^ SNL values are useful for evaluating the precision of a method (i.e., the σ_AE_ for ratios and σ_ER_ for delta values) given a certain instrument configuration and sample concentration.

Assuming that the variance for the sample and standard acquisitions are approximately equal, the SNL can be defined using the following equation for a given mass fragment of a molecule:^29^

8 (σRR13)2=(1+R13)2E×M×R13=1NM+1Nm

σ_*R*_ is the precision achievable at the SNL, ^13^ *R* is the ^13^C/^12^C ratio for a given molecule or fragment of interest, *E* is the ionization efficiency (the number of molecular or fragment ions received at the C-Trap per molecule introduced to the EI source), *M* is the number of analyte molecules introduced (the product of the number of moles introduced, *m*, and Avogadro’s number, *N*_A_), *N*_m_ is the number of minor (rare) ions, and *N*_M_ is the number of major (unsubstituted) ions. The abundance of ions produced for mass fragments can vary widely and reflect both the ionization efficiency of the molecule and the relative stability of the specific fragment versus the molecular ion. For isotope analyses, SNL thus reflects the production efficiency of individual fragment ion. Introducing δ notation (eqs 1 and 6) and simplifying the propagated errors gives the following equation:^29^

9 σδ2=(2×106)×(1+R13)2E×M×R13=(2×106)×[(1Nm)+(1NM)]

If *N*_M_ ≪ *N*_m_, which is true for carbon isotopes at natural abundance, 1/*N*_M_ can be considered negligible and can be removed from the equation. When rearranged, eq 9 allows one to calculate the minimum number of ions required to achieve a given precision for δ^13^C measurements:

10 $$N_{m}=\frac{2\times 10^{6}}{{\sigma_{\delta }}^{2}}$$

### **Fragmentation Pattern of Pyruvic Acid and Acetic Acid; Calculation of Intramolecular δ** ^**13**^ **C Values**

Representative mass spectra with the fragment carbons labeled for pyruvic acid and acetic acid are shown in Figure 2a,b.

**Figure 2:**
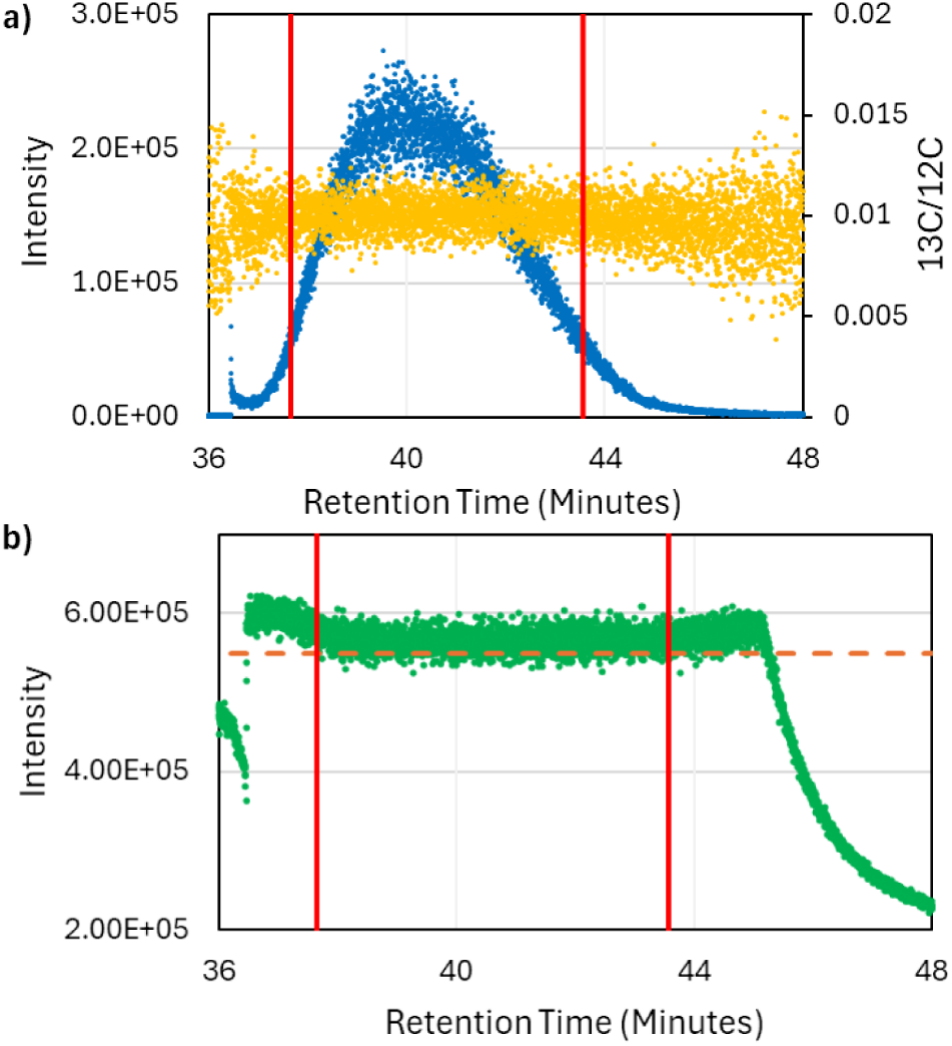
(a) Representative intensity (blue) profile of the mass 46 fragment of acetic acid delivered to the Orbitrap after it has been trapped for 30 min using the peak trapping system. Using a 20% culling threshold of the maximum intensity value (red bars) conservatively isolates the region in which the ^13^ *R* value (yellow) is most stable. (b) This is supported by the total ion count (TIC) * injection time (IT; ms) profile showing that region is in AGC control (orange dashed line).

EI-Orbitrap MS analyses of pyruvic acid labeled with ^13^C at either C1 (carboxyl) or C3 (methyl) positions confirmed the carbon atoms present in each mass fragment (Figure 2a): fragment 43 in pyruvate contains carbons C2 and C3 and the keto oxygen, whereas fragment 45 contains only the carboxyl group. Similarly, analysis of acetic acid labeled with ^13^C at the C2 (methyl) carbon confirmed the carbon atoms present in the two main fragments (Figure 2b). Fragment 43 in acetic acid comes from a loss of the −OH group from the carboxyl carbon and contains both carbon atoms. Fragment 45 contains the C1 (carboxyl) carbon (Figure 3).

**Figure 3:**
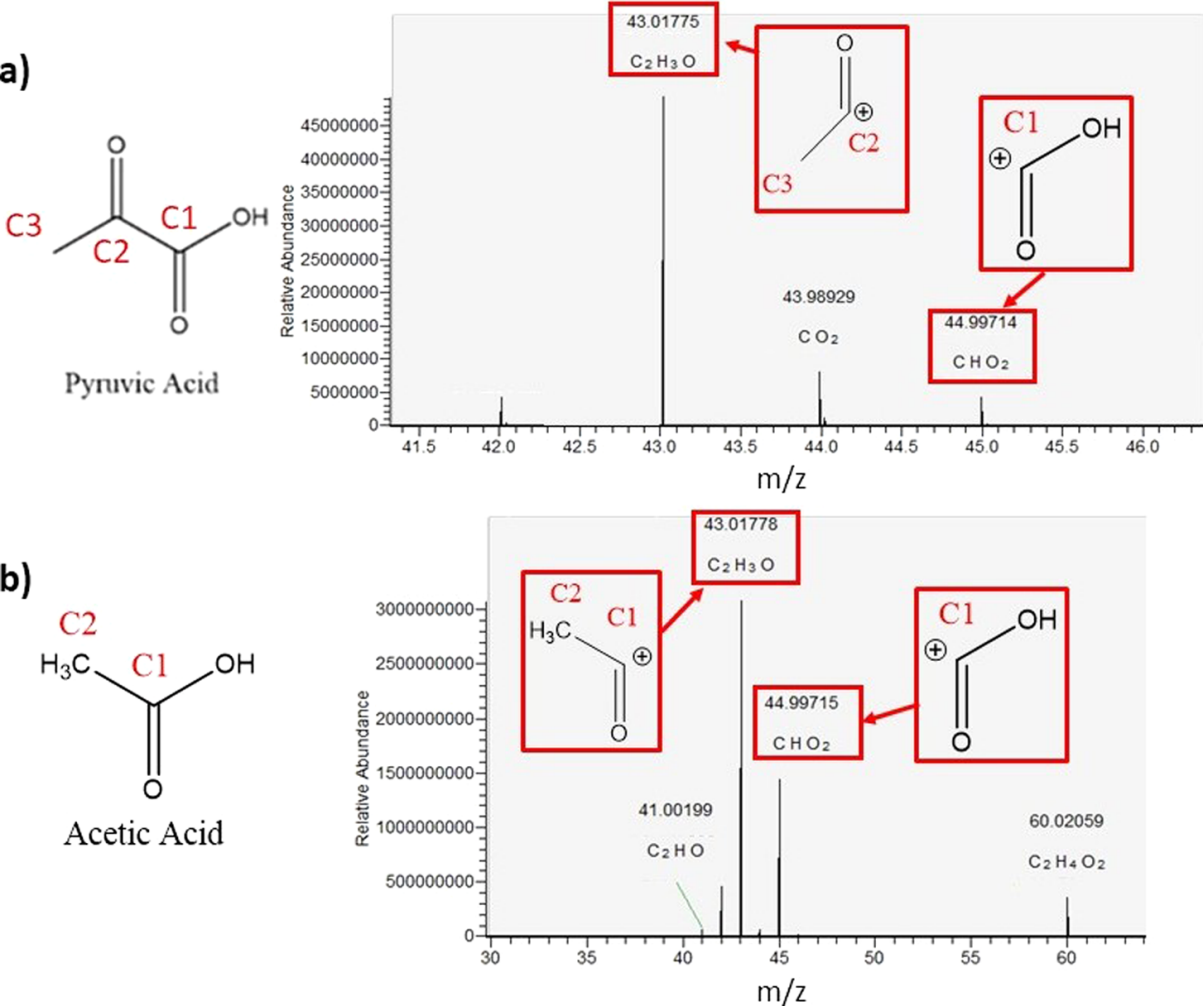
(a) Mass spectrum for pyruvic acid analyzed via the GC Orbitrap-MS system, highlighting mass fragments used for PSIA and corresponding chemical structures (red box) with the individual carbons in pyruvic acid labeled in red. (b) Mass spectrum for acetic acid analyzed via GC Orbitrap-MS, highlighting the mass fragments used for PSIA and corresponding chemical structures (red box) with the individual carbons in pyruvic acid labeled in red.

With knowledge of how both analytes fragment, it is then possible to calculate position-specific carbon isotope ratios. For pyruvic acid, the δ^13^C value of the C1 carbon and an average δ^13^C value of C2 + C3 from pyruvate are measured directly from fragments 45 and 43, respectively, and the molecular average δ^13^C value can be calculated using the ^13^ *R* of the two fragments and accounting for the number of carbons in each fragment:^30^

11 δCmol.avg13=(2*δC4313)+(δC4513)3

For acetic acid, the molecular average and C1 δ^13^C values are measured directly from fragments 43 and 45, respectively, and the C2 δ^13^C value is calculated:

12 δCC213=(2×δC4313)−(δC4513)

### Accuracy of Isotope Ratio Results and Error Propagation

To estimate accuracy, the Orbitrap δ^13^C values were compared to measurements of the same samples using previously described complementary methods. To quantify the relative accuracy of the Orbitrap measurements, a root-mean-square deviation (RMSD) was calculated between the Orbitrap MS and each complementary instrument measurement and reported in the associated figures.

Molecular average δ^13^C values for each pyruvic acid and acetic acid sample were also analyzed via conventional GC-IRMS at Penn State. Acetic acid standards previously characterized by the methods described in Yamada et al.^27^ were also reanalyzed via GC EI Orbitrap.

Sample isotope measurements are reported relative to the lab standard, and measurements of both are associated with a degree of uncertainty (i.e., standard errors). Each measurement’s uncertainty is then propagated in quadrature across all subsequent calculations that rely on its value and is reported as the ratio’s acquisition error, σ_AE_ when comparing average ^13^ *R* values, or its experimental reproducibility, σ_ER_ when comparing standard-sample acquisitions. Assuming uncorrelated and normally distributed errors in both the calculated and known values, the propagated errors are determined by the standard methods described in Bevington & Robinson.^31^ If *y = f­(a,b,···)*, the independent, normally distributed errors in *a* and *b* (σ_*a*_ and σ_*b*_, respectively) are propagated to give the uncertainty in *y* (σ_*y*_):

13 $$\sigma_{y}=\sqrt{{\left(\frac{\partial_{y}}{\partial_{a}}\times \sigma_{a}\right)}^{2}+{\left(\frac{\partial_{y}}{\partial_{b}}\times \sigma_{b}\right)}^{2}}$$

The relevant calculations for σ_AE_ used for each step in finding the propagated error associated with the position-specific δ^13^C_std_ values are reported in the Supporting Information.

## Results

### Method Optimization and Validation

Optimum sample injection amounts are dictated by the abundance of the least abundant isotopologue of interest. For both PA and AA, this is the M + 1 isotopologue of the 45 *m*/*z* fragment ^13^CO_2_H^+^; at 46.00053 *m*/*z*), whose maximum intensities were ∼0.10 and ∼0.60% of the fragment 43 intensity (the most abundant fragment), respectively. ∼100 nmols of analyte were sufficient for precise (<2.0‰ σ_ER_ on average for both pyruvic acid and acetic acid) intramolecular isotope measurements of both acids. When analyzed in triplicate, our δ^13^C measurements achieved σ_AE_ of <0.2‰ for isotope ratios of both pyruvic acid fragments, with experimental errors (σ_ER_) ranging from 0.57 to 2.83‰ (Table S4). Errors for both fragments of acetic acid achieved σ_AE_ < 0.12‰ and σ_ER_ ranging from 0.05 to 3.37‰ (Table S5).

### Accuracy of Intramolecular δ^13^C Measurements of Pyruvic Acid on the GC Orbitrap MS

All analyses of pyruvic acid on the GC-Orbitrap MS were conducted with ∼100 nmol sample injections. The results are summarized in Tables 1, S4, and Figure 4a.

**Figure 4:**
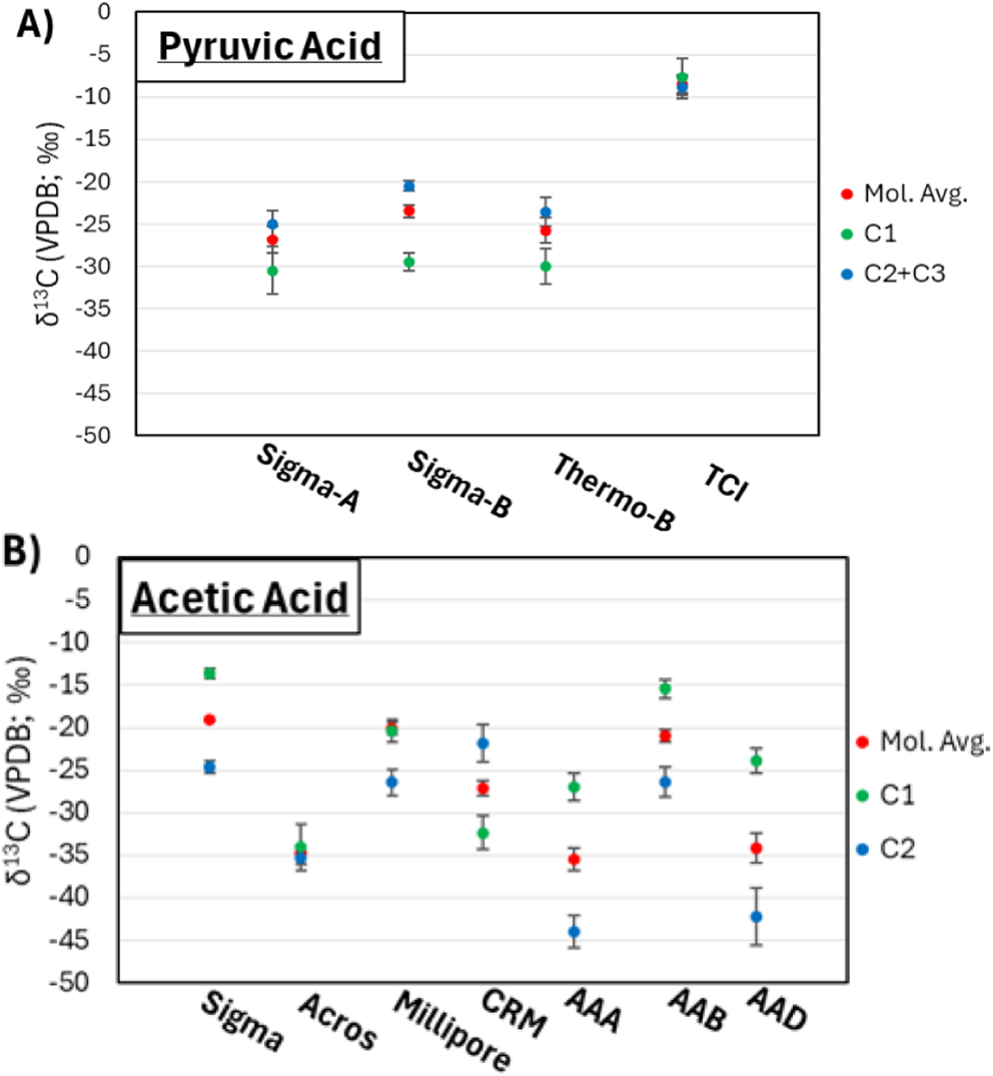
(a) Intramolecular δ^13^C_VPDB_ (‰) differences between pyruvic acid standards as measured by GC-Orbitrap MS. (b) Intramolecular δ^13^C_VPDB_ (‰) differences between acetic acid standards. Experimental error (σ_ER_) values given as error bars.

**Table 1:** Results of GC Orbitrap MS Measurements Relative to Complementary Measurements on the GC IRMS and GC-py-GC IRMS

Pyruvic Acid				
	GC-IRMS	GC-py-GC IRMS		
Carbon position	Molecular average	Molecular average	C1	C2 + C3
Range of offset (‰)	07–4.1	1.2–3.8	0.6–3.6	21–3.9
Average offset (‰)	2.3	2.2	2.1	2.7
RMSD (‰)	2.6	2.5	2.5	2.8

Acetic acid				
	GC-IRMS	GC-py-GC IRMS		
Carbon position	Molecular average	Molecular average	C1	C2
Range of offset (‰)	0.5–3.9	1.4–3.2	0.1–2.8	2.8–6.5
Average offset (‰)	2.1	2.1	1.6	4.6
RMSD (‰)	2.3	2.2	1.9	4.9

The molecular average δ^13^C_VPDB_ values of the PA standards measured on the Orbitrap differed from the 252 GC-IRMS values from a range of 0.7‰ to 4.1‰ with an average net offset of 2.3‰ (Figure 5a). Comparison of the Orbitrap MS measurements relative to the GC-IRMS method yielded an RMSD of 2.6‰.

**Figure 5:**
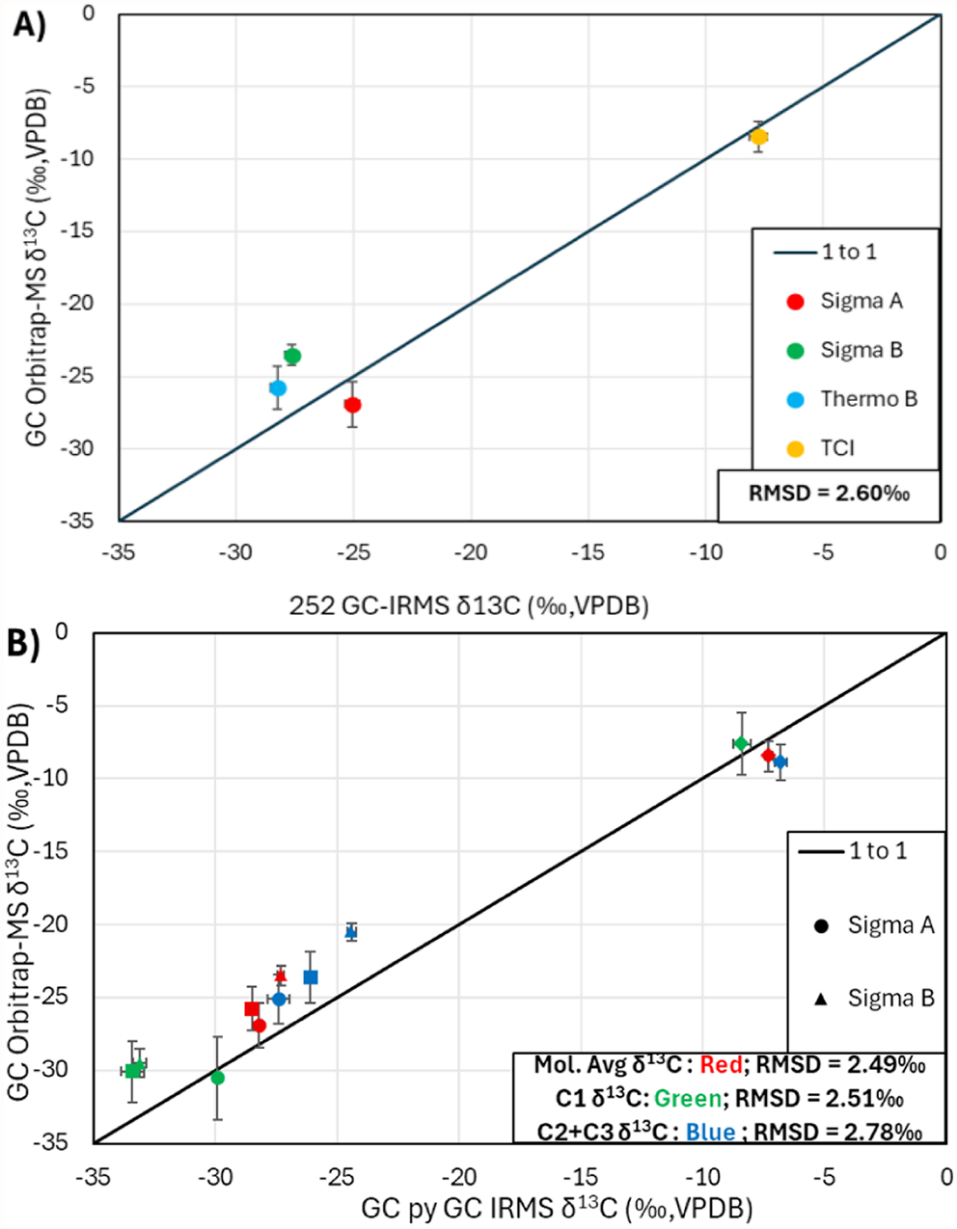
(a) Molecular average δ^13^C_VPDB_ of pyruvic acid standards measured by 252 GC-IRMS compared to the GC Orbitrap MS measurements. Experimental error (σ_ER_) values given as error bars. (b) Position-specific δ^13^C_VPDB_ of pyruvate standards measured via GC Orbitrap-MS compared to the GC-py-GC IRMS measurements. Molecular average δ^13^C_VPDB_ measurements are shown with red symbols, C1 δ^13^C_VPDB_ with blue, and the average C2 + C3 δ^13^C_VPDB_ with green. Analyses of different standards are denoted by shape. Experimental error (σ_ER_) values given as error bars.

The two position-specific methods (GC Orbitrap-MS vs GC-py-GC IRMS) were then compared by analyzing the same pyruvate samples (Table S4 and Figure 5b). The pyruvate molecular average δ^13^C_VPDB_ measurements differed from 1.2‰ to 3.8‰ with an average offset of 2.2‰ between the two methods. Differences in the average C2 + C3 δ^13^C_VPDB_ values of pyruvate ranged from 2.1 to 3.9‰ with an average offset of 2.7‰. The δ^13^C_VPDB_ value of C1 differed from 0.6‰ to 3.6‰ with an average offset of 2.1‰. Orbitrap MS measurements of the molecular average, C1, and C2 + C3 δ^13^C_VPDB_ values relative to the GC-py-GC IRMS method yielded RMSD values of 2.5‰, 2.5‰, and 2.8‰, respectively.

### Accuracy of Intramolecular δ^13^C Measurements of Acetic Acid on the GC Orbitrap MS

Aqueous acetic acid samples were measured on the GC-Orbitrap MS and Thermo Finnigan MAT 252 GC-IRMS. Analyses on the GC-Orbitrap MS were conducted with ∼100 nmol sample injections and reported relative to VPDB (δ^13^C_VPDB_). The results for the acetic acid analyses on both instruments are summarized in Tables 1, S5 and Figure 4b.

The molecular average δ^13^C value measured via GC-IRMS was reported relative to VPDB (Figure 6a). Differences between the acetic acid molecular averages measured on the Orbitrap method and GC-IRMS values ranged from 0.5‰ to 3.9‰ with an average offset of 2.1‰ and an RMSD of 2.3‰. Position-specific measurements made on the Orbitrap were then compared to previously characterized samples from Tokyo Tech (Figure 6b).^27^ Differences in the molecular average δ^13^C measurements between the Orbitrap and published values^27^ differed from a range of 1.4‰ to 3.2‰ with an average offset of 2.1‰. The C1 δ^13^C value differed from 0.1‰ to 2.8‰ with an average offset of 1.6‰. The δ^13^C value of C2 ranged from 2.8‰ to 6.5‰ with an average offset of 4.6‰. Orbitrap MS measurements of the molecular average, C1, and C2 δ^13^C_VPDB_ value relative to the GC-py-GC IRMS method yielded RMSD values of 2.2‰, 1.9‰, and 4.9‰, respectively.

**Figure 6:**
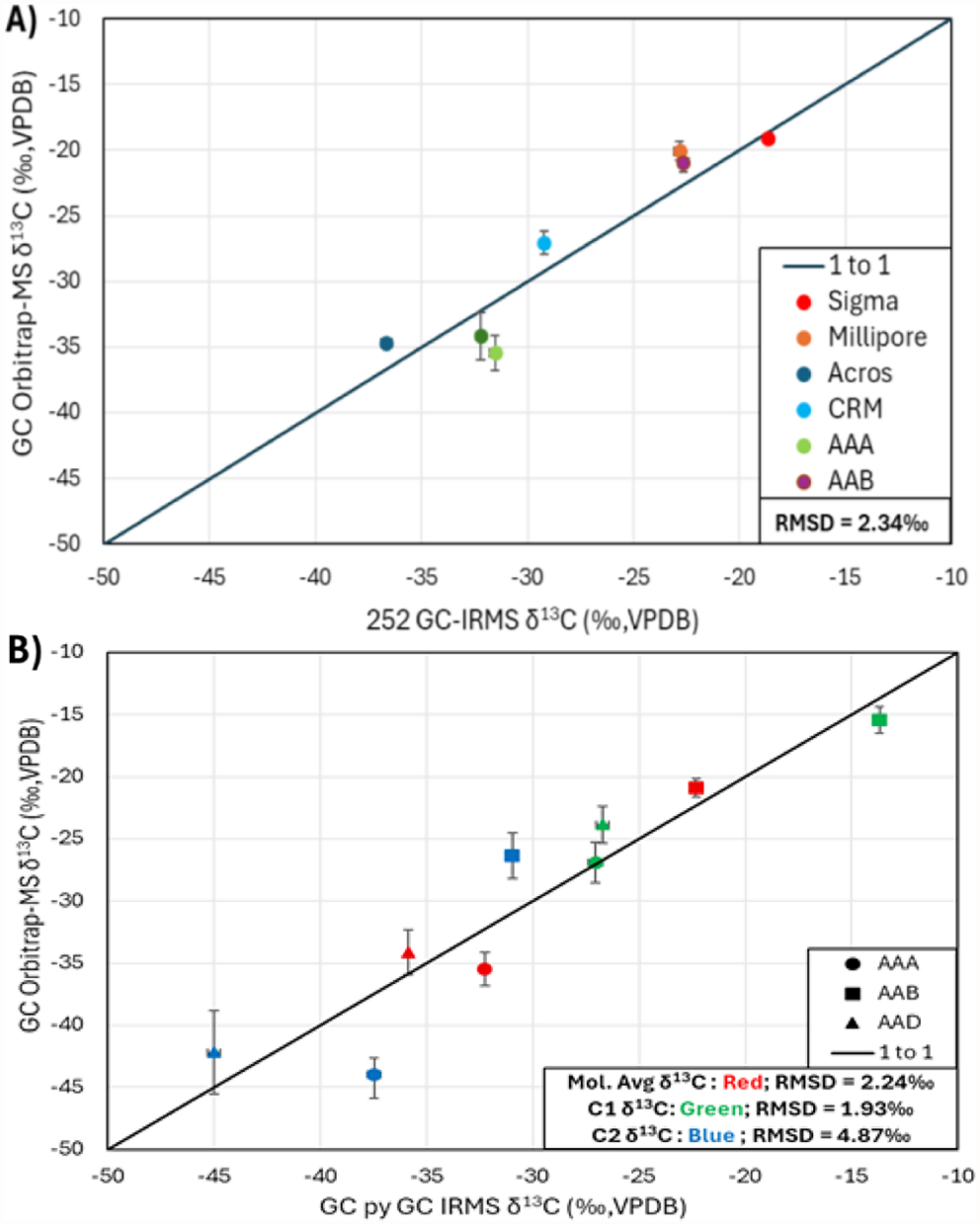
(a) Molecular average δ^13^C_VPDB_ of acetic acid standards measured by 252 GC-IRMS compared to the Orbitrap measurements. Experimental error (σ_ER_) values given as error bars. (b) Position-specific δ^13^C_VPDB_ of acetate standards measured by GC Orbitrap-MS compared to the GC-py-GC IRMS measurements as an estimate of the accuracy of the former method. Molecular average δ^13^C_VPDB_ measurements are shown with red symbols, C1 δ^13^C_VPDB_ with blue, and the average C2 + C3 δ^13^C_VPDB_ with green. Analyses of different standards are denoted by shape, and experimental error (σ_ER_) values given as error bars.

## Discussion

The data presented here describe the first measurements of intramolecular δ^13^C patterns of pyruvic and acetic acid on an GC Orbitrap MS system. Peak capture enabled by modifications in the GC flow path combined with the use of a polar GC column allows for measurement of isotope ratios at nanomolar injections of analyte without prior chemical derivation steps and offline, labor-intensive chemical degradation steps that were necessary in previous studies.^2–4^ Position-specific δ^13^C measurements were possible with experimental errors (σ_ER_) ranging from 0.57 to 2.83‰ for PA (Table S4) and from 0.05 to 3.4 ‰ for acetic acid (Table S5).

### Limitations on Precision and Accuracy

Differences in isotope values reported here for the GC Orbitrap MS method and conventional methods can be attributed to several factors. For both compounds, the low abundance of the F_45_ ion fragment results in a higher associated error compared to the F_43_ fragment. The F_45_ fragments are used to determine isotope ratios for the molecular average of pyruvic acid and C2 of acetic acid, and these are each used in subsequent calculations. In all such calculations, the lowest abundant fragment limits the accuracy and precision of the isotope values reported using the Orbitrap method.

When the analyte concentration is not the limiting factor for improving the measurement precision, reducing the mass resolution or increasing the AGC target for high concentration samples may help with increasing the number of scans registered for the least abundant fragment and its M + 1 isotopologue.^22^ Further decreasing the He flow rate upon reintroducing the sample loop to the main flow path or installation of a higher volume sample loop can also potentially increase width of the observed peak and subsequent time an analyte is monitored. Higher volume sample loops can further diffuse trapped analytes. However, this will require adjustments to the Orbitrap MS settings to account for the overall lower ion intensities and to ensure AGC control is reached.

However, for analyses where more than one elemental isotopologue is monitored, lowered mass resolutions may prevent the separation of singly substituted isotopologues (i.e., ^13^C from ^2^H or ^17^O), potentially affecting the accuracy of ^13^ *R* measurements. For example, a resolution of at least >53,490 is required to differentiate between the ^13^C (46.00101 amu) and ^17^O isotopomers (46.00187 amu) of the 45 *m*/*z* fragment (*m*/Δ*m* = 46.00101/(46.00187 – 46.00101) = 53,489.55). Therefore, it is important to find the optimal run settings for each compound and isotope systems of interest.

While efforts were made to ensure the modified GC Orbitrap MS system was airtight, there is some background CO_2_ present in the sample loop system when analytes are reintroduced to the main gas flow. Both acetic acid and pyruvic acid produce a CO_2_ fragment when introduced to the EI source. Hofmann et al.^32^ found that the accuracy and precision of isotope measurements of individual components within a mixture with similar mass fragmentation patterns measured via GC Orbitrap MS can be affected by the presence of potential “contaminant ion peaks,” which are defined in their work as fragment ions from other molecules that are unrelated to the fragment ions of interest, in the analyzed mass window. As the same isotopic information for the C1 carboxyl carbon can be measured using an adjacent peak (the 44 *m*/*z* CHO_2_ fragment), the CO_2_ fragment was not used for calculating intramolecular isotope ratios to avoid any effects from background CO_2_ present in the system. Additionally, as the background CO_2_ peak was consistently less than 20% of the analyte CO_2_ peak, we did not expect any significant effect of the background CO_2_ presence on the isotope ratios measured by the Orbitrap.

### Pyruvic Acid Stability

In aqueous solutions, pyruvic acid can undergo aldol condensation. The resulting dimer, parapyruvate, can co-occur as the dicarboxylic acid and as a cyclic lactone intermediate.^24^ Pyruvic acid is relatively stable at −20 °C, but at higher temperatures it can slowly decarboxylate to acetic acid.^33^ Dimerization and decarboxylation reactions involve a change from sp^3^ to sp^2^ hybridization for bond formation and carbon–carbon bond breakage, respectively, and either can produce isotope fractionation.^34^ Pure and unacidified pyruvic acid was shipped to Tokyo Tech, and it may have partially degraded to the pyruvic acid dimer during shipment. This could have been accompanied by decarboxylation reactions to form acetic acid or other reaction products over time. Dimerization could generate a pyruvic acid monomer depleted in the δ^13^C values at the C2 and C3 carbon positions. Consistent with this suggestion, Tokyo Tech measurements were depleted compared to Orbitrap measurements, which involved exclusively freshly made solutions. Multilaboratory, timely analyses of temperature-controlled aliquots of acidified organic acid standards will help determine whether temperature-related degradation caused the apparent fractionation between the two position-specific isotope analysis methods, or the differences are due to instrument-specific fractionation.

### Shot Noise Limits (SNLs) and Fragment-Specific Ionization Efficiencies (*E*_frag_) for Pyruvic Acid and Acetic Acid Intramolecular δ^13^C Measurements

SNLs and ionization efficiencies for both fragments were calculated (*E*_frag_) based on the ∼100 nmol injections used for acetic acid and pyruvic acid in this study. Table S6 lists the parameters used to calculate *E*_frag_ and SNLs, which were chosen from a single representative acquisition of acetic acid or pyruvic acid. Acquisitions of acetic acid achieved errors (σ_AE;_ calculated as the relative standard error of a given fragment’s average ^13^ *R* from a single acquisition) that were 10.52–11.92 times higher than SNL, with *E*_43_ = 2.22 × 10^–7^ and *E*_45_ = 4.88 × 10^–8^. Acquisitions of pyruvic acid showed σ_AE_ errors that were 45–48 times the SNL, with *E*_43_ = 1.76 × 10^–6^ and *E*_45_ = 1.82 × 10^–7^. When reintroduced into the GC carrier flow after being trapped in the sample loop, the acetic acid acquisitions used in this study tended to have a wider, more diffuse peak shape when compared to the pyruvic acid runs. As a result, acetic acid acquisitions had more scans associated with each injection, which contributed to a lower overall error compared to pyruvic acid despite its lower E for both fragments.

Given that *E* has been calculated for both fragments, a rearrangement of eq 10 also allows for an estimate of the theoretical minimum amount of analyte (*m*) required for a given precision:

14 $$m\;\left(\mathrm{moles}\right)=\left(\left(2\times 10^{6}\right)\times \frac{{(1+R)}^{2}}{{\sigma_{\delta }}^{2}ER}\right)/N_{A}$$

For example, with *E*_45, AA_ = 4.88 × 10^–8^, achieving a ±1‰ precision for the 45 *m*/*z* fragment in acetic acid requires a minimum injection of 72 nanomoles. The corresponding *E*_45_ for pyruvic acid is an order of magnitude greater at 1.83 × 10^–7^, suggesting an injection of at least 1 nanomole should be sufficient for 1‰ precision. As demonstrated in our data (Tables S4 and S5), the required amount of injected analyte for that level of precision is higher in practice, reflecting aspects other than the stochastic nature of the ion current dictate the achievable precision of our method.

We provide here a benchmark for future Orbitrap users interested in calculating fragment-specific SNLs and *E*_frag_ to assist in their efforts to optimize methods for analysis of isotopes. Here, while the theoretical fragment-specific SNL calculated via ion counting statistics^6,29^ was not met by our current configuration, the precision achieved for both analytes is still sufficient to study intramolecular fractionations due to both biological and abiotic phenomena (see below). Future research efforts using this method will further refine instrument parameters and sample preparation to improve experimental errors toward the SNL.

### Summary and Future Directions

The accuracy and precision achieved using the Orbitrap holds promise for applications in future studies. For example, biological processes that involve enzyme activity can impart intramolecular kinetic isotope effects (KIE) specific to the region where a bond may be formed or broken. The conversion of pyruvate to acetyl-CoA (and subsequent release of the C1 carbon of pyruvate as CO_2_) via pyruvate dehydrogenase has been found to impart up to a 23‰ kinetic isotope effect (KIE) on the C1 position of acetyl-CoA and the C2 position of residual pyruvic acid, and a 3‰ KIE for the C2 position of acetyl-CoA and the C3 position of residual pyruvic acid.^35^ However, intramolecular isotope patterns measured in nature may not solely reflect the full isotope effect of enzymatic processes: substrate availability or changing environmental conditions can also affect the flux of carbon through reaction pathways. For example, carbon isotope measurements of porewater acetate collected from anoxic marine sediments in Cape Lookout Bight, North Carolina showed a depletion of 7–14‰ in the methyl carbon relative to the carboxyl carbon of acetate. These values reflect not only enzymatic KIE but also the effect of acetate cycling on measured intramolecular isotope patterns.^36^

Additionally, carbon isotope patterns for monocarboxylic acids sourced from interstellar precursor carbon pools are predicted to vary over tens of permil relative to the starting precursors.^30^ In both cases, the precision achieved by the method described in this paper would be sufficient to study the intramolecular isotopic patterns imparted on small organic acids from both biological and abiotic synthesis pathways.

Environmental samples may have lower concentrations than those analyzed in this study. For example, fracture waters collected from the Canadian Shield at Kidd Creek Mine exhibit millimolar concentrations of formate and acetate and can be considered to be a high-concentration endmember for porewater samples.^19^ While organics synthesized in extraterrestrial environments are known to show dramatic (10 s–100 s of ‰) isotopic differences between carbon positions,^22,30^ the concentration of extractable carboxylic acids such as acetic acid from carbonaceous meteorites top out at about 100 μmol/g of meteorite sample.^37^

In these cases, additional steps such as off-line sample concentration,^25^ lowering the split flow of the sample inlet, lowering the AGC target, derivatization to improve ionization efficiencies in the mass spectrometer, or running separate acquisitions to capture only the ^13^C isotopologue (M + 1 experiments)^11^ to ensure AGC control is met during the eluted peak may be required for accurate and precise isotope measurements. The presence of contaminant ions within the analysis window can potentially be addressed by narrowing the mass window, matching the matrix of the sample and standard, or adjusting the mass resolution to resolve various adducts may help to achieve better precision of isotope measurements within a mixture such as those that may be found in environmental samples.^32^

## Conclusion

The peak trapping system installed on the GC-Orbitrap MS located at Penn State University offers an alternative method to those published previously.^6^ A novel method was developed using the peak trapping system for measuring the intramolecular δ^13^C values of two astrobiologically relevant organic acids, pyruvic acid and acetic acid. Provided that the injection amounts and analysis methods of both standard and sample are similar, a 100 nmol injection of analyte yields enough ion scans for precise and accurate intramolecular δ^13^C measurements of both pyruvic acid (σ_AE_ < 0.2‰; σ_ER_ < 2.8‰) and acetic acid (σ_AE_ < 0.12‰; σ_ER_ < 3.4‰). The main source of error for intramolecular δ^13^C measurements on the Orbitrap is the low number of observed fragment ions for the least abundant isotopologue, highlighting the analytical difficulty in accurately measuring the isotope value of reactive organic acids. This is also reflected in our SNL and *E*_frag_ calculations for both pyruvic acid and acetic acid, which show low ionization efficiencies and modest experimental errors relative to shot noise. Narrowing the mass window for each run, matching the matrix of the sample and standard, or adjusting the mass resolution to resolve various adducts may help to achieve better precision of isotope measurements, but the optimal run conditions will vary with the ionization properties of the analyte in question.

## Supplementary Material



## Notes

Preliminary trapping experiment methods and results with tetradecane on the gas chromatography Orbitrap mass spectrometer (GC Orbitrap MS) with sample loop installed; absolute intensity of peaks trapped in the sample loop for certain amounts of time before release into the mass spectrometer ion source; Table S1: ^13^ *R*s and associated errors for tetradecane trapped for different times; Table S2: position-specific δ^13^C values of pyruvic acid standards measured via GC py-GC IRMS at Tokyo Tech, reported relative to Vienna Pee Dee Belemnite (VPDB); error propagation steps involved in calculating position-specific δ^13^C values measured via GC-Orbitrap MS; Table S3: glossary defining key variables and abbreviations used for calculating propagated errors; Table S4: molecular average and position-specific δ^13^C_VPDB_ (‰) of purchased pyruvic acid standards, including the associated acquisition errors (σ_AE_) and experimental errors (σ_ER_); Table S5: molecular average and position-specific δ^13^C_VPDB_ (‰) of acetic acid standards, with acquisition errors (σ_AE_) and experimental errors (σ_ER_) reported for all methods; analysis of pyruvic acid using the GC py-GC IRMS method at Tokyo TechAnalysis of Aqueous Acids using the 252 GC-IRMS, including sealed tube pyrolysis; Table S5: parameters used from representative acquisitions of pyruvic acid (PA) and acetic acid (AA) to calculate fragment-specific ionization efficiencies (*E*_frag_) and shot noise limits (SNLs) (PDF)
