# Supplementary material for: Molecular Average and Intramolecular δ13C Measurements of Pyruvic Acid and Acetic Acid Using Gas Chromatography Orbitrap Mass Spectrometry (GC Orbitrap-MS)
Source: Anal Chem. 2025 Aug 26;97(35):19047–56. doi: 10.1021/acs.analchem.5c02194 (PMC12424967; doi:10.1021/acs.analchem.5c02194)
Supplement: Supplementary file 1 [file ac5c02194_si_001.pdf]

Supplementary Information

Molecular average and Intramolecular δ13C Measurements of Pyruvic Acid and Acetic Acid using Gas Chromatography Orbitrap Mass Spectrometry (GC Orbitrap-MS)

Youki J. Sato[1]*, Allison A. Baczynski[1], Hao Xie[2], Alexis Gilbert[3], and Katherine H. Freeman[1]

[1]Astrobiology Center for Isotopologue Research, Department of Geosciences, Pennsylvania State University, University Park, Pennsylvania, 16802, United States

[2]International Center for Isotope Effects Research, Nanjing University, Nanjing, 210023, China

[3]Institute for Marine and Atmospheric Research Utrecht (IMAU), Utrecht University, Utrecht, 3526 KV, The Netherlands

***Corresponding Author Email: yws5168@psu.edu**

Table of Contents:

Preliminary trapping experiment methods and results with tetradecane on the gas chromatography Orbitrap mass spectrometer (GC Orbitrap MS) with sample loop installed.

Figure S1: Absolute intensity of peaks trapped in the sample loop for certain amounts of time before release into the mass spectrometer ion source.

Table S1: ^{13}Rs and associated errors for tetradecane trapped for different times.

Table S2: Position-specific δ^{13}C values of pyruvic acid standards measured via GC py-GC IRMS at Tokyo Tech, reported relative to Vienna Pee Dee Belemnite (VPDB).

Error propagation steps involved in calculating position-specific δ^{13}C values measured via GC-Orbitrap MS.

Table S3. Glossary defining key variables and abbreviations used for calculating propagated errors.

Table S4. Molecular average and position-specific δ^{13}CVPDB (‰) of purchased pyruvic acid standards, including the associated acquisition errors (σ_{AE}) and experimental errors (σ_{ER}).

Table S5. Molecular average and position-specific $\delta^{13}C_{VPDB}$ (‰) of acetic acid standards, with acquisition errors (σ_{AE}) and experimental errors (σ_{ER}) reported for all methods.

Analysis of Pyruvic Acid using the GC py-GC IRMS Method at Tokyo Tech

Analysis of Aqueous Acids using the 252 GC-IRMS, Including Sealed Tube Pyrolysis

Table S5. Parameters used from representative acquisitions of pyruvic acid (PA) and acetic acid (AA) to calculate fragment-specific ionization efficiencies (E_{frag}) and shot noise limits (SNLs).

Supplementary References

Supplementary Information

Preliminary trapping experiment methods and results with tetradecane on the gas chromatography Orbitrap mass spectrometer (GC Orbitrap MS) with sample loop installed.

Trapping experiments were conducted using nanomole (50 ng/µL) injections of tetradecane dissolved in hexane to confirm whether the sample loop system in the GC flow path would be effective to increase the analyte observation time. Tetradecane was chosen because it efficiently ionizes in the ion source of the Orbitrap mass spectrometer, yielding intense peaks without prior derivatization on nonpolar GC columns. The C_5H_{10} (70.1 m/z) fragment of tetradecane was analyzed for its ^{13}R value after either: allowing it to flow directly from the GC column to the Orbitrap, routing the GC flow path through the sample loop on the way to the Orbitrap without trapping, or after being trapped for 15, 30, 60, or 180 minutes in the sample loop prior to reintroduction to the Orbitrap ion source.

Analyses were made using a Thermo Scientific (Thermo) Q-Exactive Orbitrap coupled to a Thermo Trace 1310 GC with a liquid injection port. The GC column was a 30 m x 0.25 mm x 0.25 µm thickness Restek Rtx®-5 with Integra-guard. The split/splitless sample inlet was set to a split configuration and kept at 200°C with a split flow of 20 mL/min. The helium flow was kept at 1 mL/min for the duration of the run. The gas chromatography oven temperature was programmed to start at 60ºC, held for 6 minutes, then increased to 140°C at 10°C/min. This temperature was then held for the appropriate amount of time for the trapping test to keep the analyte in the gas phase during expansion within the sample loop before delivery to the ion source. The temperature was then ramped to 260°C at a rate of 40°C/min to clear the column for the next analysis. The ion source and transfer line to the mass spectrometer were kept constant at 280°C and 250°C, respectively. The operating parameters for the Orbitrap MS were as follows: 1 microscans per cycle, a mass window of 62-78 m/z to capture the 70 m/z mass fragment and associated isotopologue, an AGC target of $1x10^6$ ions, a maximum injection time of 120 ms, and a mass resolution of 60,000. Data were collected for the first 8-14 minutes, then reactivated one minute before reintroduction of the sample loop flow to the system, until the end of the run. The stainless-steel sample loop and valve system are described in the Materials and Methods section of the main text.

Tetradecane released from the sample loop yielded an elongated peak on the scale of minutes as opposed to the un-trapped peak, which was tenths of a second long (Figure S1). As a result of these increased observation times, relative standard errors calculated for the ^{13}R value of the C_5H_{10} fragment of tetradecane improved from 4.81‰ for the un-trapped peak to between 0.29-0.39‰ post-trap (Table S1).

Figure S1: Absolute intensity of peaks for the C_5H_{11} mass fragment of tetradecane trapped in the sample loop for certain amounts of time before release into the mass spectrometer ion source.

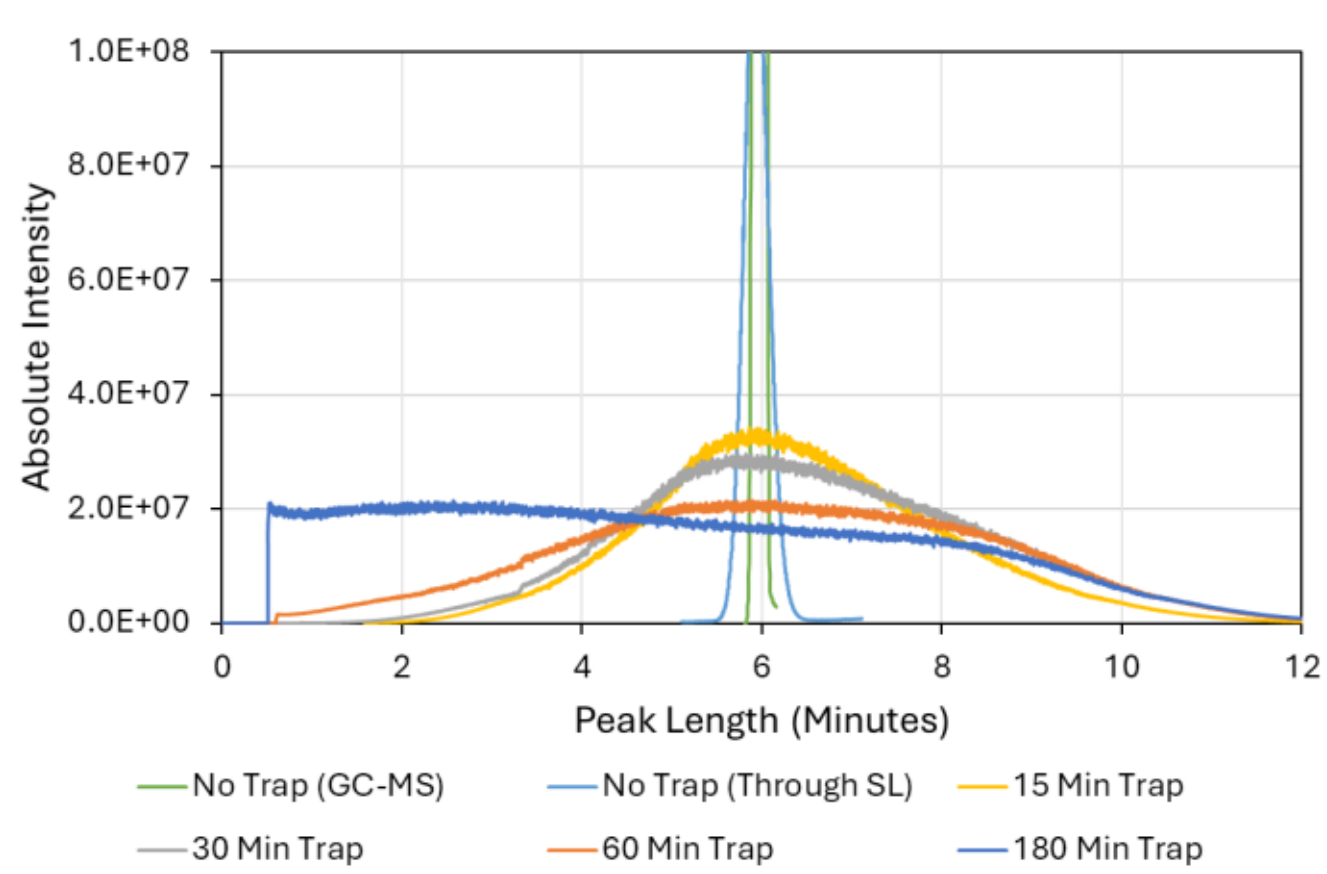

Table S1: ^{13}Rs and associated errors for the C_5H_{11} mass fragment of tetradecane trapped for different times.

Trap Time (Min.)	Average ^{13}R	Std. Error	Relative Std. Error (‰)
GC-MS (No Trap)	0.06071	2.92E-04	4.81
Thru SL (No Trap)	0.05675	1.56E-04	2.75
15	0.06095	2.36E-05	0.39
30	0.06095	2.12E-05	0.35
60	0.06103	1.90E-05	0.31
180	0.06077	1.77E-05	0.29

Table S2. Position-specific δ^{13}C of pyruvic acid standards measured via GC py-GC IRMS at Tokyo Tech, reported relative to Vienna Pee Dee Belemnite (VPDB).

	δ^{13}C (‰; VPDB)			
Company	C1 (Carboxyl)	C2 (Ketone)	C3 (Methyl)	Bulk
TCI (6/2022)	0.0 ± 0.1	-7.9 ± 0.6	-8.7 ± 1.4	-5.5 ± 0.3
TCI (5/2023)	-8.4 ± 1.0	-8.0 ± 0.5	-5.6 ± 0.4	-7.3 ± 0.2
Sigma (A)	-29.9 ± 0.2	-24.5 ± 0.8	-30.3 ± 0.8	-28.2 ± 0.2
Sigma (B)	-33.1 ± 0.8	-22.9 ± 0.2	-25.9 ± 0.4	-27.3 ± 0.1
Thermo (B)	-33.4 ± 1.4	-25.1 ± 0.4	-27.1 ± 0.3	-28.5 ± 0.2
Acros	-30.9 ± 0.1	-26.2 ± 0.6	-29.3 ± 0.5	-28.8 ± 0.1

Error propagation steps involved in calculating position-specific δ^{13}C values measured via GC-Orbitrap MS:

See Table S3 for a glossary of abbreviations used for calculating propagated errors. Assuming uncorrelated and normally distributed errors in both the calculated and known values, the propagated errors can be determined by the sum of squares methods as described in Bevington & Robinson[1]. If *y=f(a,b…)*, the independent, normally distributed errors in *a* and *b (*σ_a and σ_b, respectively*)* can be propagated to give the uncertainty in *y (*σ_y*):*

$$\sigma_y = \sqrt{(\frac{\partial y}{\partial a} * \sigma_a)^2 + (\frac{\partial y}{\partial b} * \sigma_b)^2 \dots} \quad \text{(S1)}$$

Error Propagation for average ^{13}R values of a mass fragment

^{13}R values for each mass fragment of interest are calculated as the ratio of ion counts of all singly substituted ^{13}C isotopologues for a given fragment vs. the unsubstituted ^{12}C isotopologue for each scan (the span of time in which the Orbitrap observes a packet of ions) The average ^{13}R across all scans (typically in the thousands) is averaged into a single value:

$$Avg\ ^{13}R_{frag} = \frac{\sum {}^{13}R_{frag}}{n} \quad \text{(S2)}$$

Next, relative standard errors associated with the average ^{13}R measurement for all replicate peaks released from the sample loop are propagated to an overall average ^{13}R value error for each mass fragment using the following equation:

$$\sigma_{\mathrm{Avg}\ ^{13}\mathrm{R}_{frag}} = \sqrt{(\sigma_{^{13}Rfrag,a} \times \left(\frac{\partial(Avg\ ^{13}\mathrm{R}_{frag})}{\partial(^{13}R_{frag,a})}\right))^2 + (\sigma_{^{13}R_{frag,b}} \times \left(\frac{\partial(Avg\ ^{13}\mathrm{R}_{frag})}{\partial(^{13}R_{frag,b})}\right))^2 \ldots} \quad \text{(S3)}$$

Equation S3 simplifies to Equation S4 below. This error is reported as the experimental reproducibility (σ_{ER}) of replicate ^{13}R measurements for a given mass fragment of the same sample:

$$\sigma_{\mathrm{Avg}\ ^{13}\mathrm{R}_{frag}} = \sqrt{\sigma_{^{13}Rfrag,a} \times \left(\frac{1}{n}\right))^2 + (\sigma_{^{13}Rfrag,b} \times \left(\frac{1}{n}\right))^2 \ldots}$$

$$\sigma_{\mathrm{Avg}\ ^{13}\mathrm{R}_{frag}} = \sqrt{(^{\sigma_{^{13}Rfrag,a}}/_{n})^2 + (^{\sigma_{^{13}Rfrag,b}}/_{n})^2 \ldots} \quad \text{(S4)}$$

Error Propagation for calculating ^{13}R relative to Vienna Pee Dee Belemnite (VPDB)

Average sample ^{13}R values measured on the Orbitrap are then converted to the VPDB scale, where the corresponding lab standard has been measured relative to VPDB using a complementary measurement approach ($^{13}R_{sample,VPDB}$; Equation 6). If the errors associated with $^{13}R_{sample,Orbi}$, $^{13}R_{standard,Orbi}$, and $^{13}R_{standard,VPDB}$ and are uncorrelated and normally distributed, these errors are propagated to the calculated $^{13}R_{sample,VPDB}$ value using the following equation:

$$\sigma_{^{13}\mathrm{R}_{sample,VPDB}} = \sqrt{\left(\sigma_{^{13}R_{sample,Orbi}} \times \left(\frac{\partial(^{13}\mathrm{R}_{sample,VPDB})}{\partial(^{13}\mathrm{R}_{sample,Orbi}}\right)\right)^2 + \left(\sigma_{^{13}R_{standard,Orbi}} \times \left(\frac{\partial(^{13}\mathrm{R}_{sample,VPDB})}{\partial(^{13}\mathrm{R}_{standard,Orbi})}\right)\right)^2 + (\sigma_{^{13}R_{standard,VPDB}} \times \left(\frac{\partial(^{13}\mathrm{R}_{sample,VPDB})}{\partial(^{13}\mathrm{R}_{standard,VPDB})}\right))^2} \quad \text{(S5)}$$

Equation S5 simplifies to Equation S6 below. This error is reported as the acquisition error (σ_{AE}) of $^{13}R_{sample,VPDB}$ measurements, representing the relative standard error for the ^{13}R measurement averaged across all scans in a peak for each mass fragment:

$$\sigma_{\mathrm{AE},^{13}\mathrm{R}_{sample,VPDB}} = \sqrt{\left(\sigma_{^{13}R_{sample,Orbi}} \times \left(\frac{^{13}\mathrm{R}_{standard,VPDB}}{^{13}\mathrm{R}_{standard,Orbi}}\right)\right)^2 + \left(\sigma_{^{13}R_{standard,Orbi}} \times \left(-\frac{^{13}\mathrm{R}_{sample,Orbi} * {}^{13}\mathrm{R}_{standard,VPDB}}{^{13}\mathrm{R}_{standard,Orbi}{}^2}\right)\right)^2 + (\sigma_{^{13}R_{standard,VPDB}} \times \left(\frac{^{13}\mathrm{R}_{sample,Orbi}}{^{13}\mathrm{R}_{standard,Orbi}}\right))^2} \quad \text{(S6)}$$

Error Propagation for position-specific δ^{13}C values

Position-specific isotope carbon ratios can be calculated according to the method and Equations 11 and 12 described in the paper. If the errors associated with the fragment average δ^{13}C values are uncorrelated and normally distributed, these errors are propagated to the calculated position-specific $\delta^{13}C_{TCI}$ value using the following equations:

$$\sigma_{\delta^{13}\mathrm{C}_{Mol\ Avg}} = \sqrt{(\sigma_{\delta^{13}C_{43}} \times \left(\frac{\partial(\delta^{13}\mathrm{C}_{Mol\ Avg})}{\partial(\delta^{13}C_{43})}\right))^2 + (\sigma_{\delta^{13}C_{45}} \times \left(\frac{\partial(\delta^{13}\mathrm{C}_{Mol\ Avg})}{\partial(\delta^{13}C_{45})}\right))^2} \quad \text{(S7)}$$

Using a similar treatment for acetic acid in Equation 12 gives:

$$\sigma_{\delta^{13}C_{C2}} = \sqrt{(\sigma_{\delta^{13}C_{43}} \times \left(\frac{\partial(\delta^{13}C_{C2})}{\partial(\delta^{13}C_{43})}\right))^2 + (\sigma_{\delta^{13}C_{45}} \times \left(\frac{\partial(\delta^{13}C_{C2})}{\partial(\delta^{13}C_{45})}\right))^2} \quad \text{(S8)}$$

These equations simplify to Equation S9 and S10 below, respectively. These errors are reported as the experimental reproducibility (σ_{ER}) of position-specific $\delta^{13}C$ measurements for replicate standard-sample acquisitions:

$$\sigma_{\delta^{13}C_{Mol\,Avg}} = \sqrt{(\sigma_{\delta^{13}C_{43}} \times \left(\frac{2}{3}\right))^2 + (\sigma_{\delta^{13}C_{45}} \times \left(\frac{1}{3}\right))^2} \quad \text{(S9)}$$

$$\sigma_{\delta^{13}C_{C2}} = \sqrt{(\sigma_{\delta^{13}C_{43}} \times (2))^2 + (\sigma_{\delta^{13}C_{45}} \times (-1))^2} \quad \text{(S10)}$$

Table S3. Glossary defining key terms and variables used for calculating propagated errors.

**Term/Abbreviation**	**Definition**
a,b,c….	Independent measurements of the same sample peak or mass fragment
n	Number of replicate measurements combined for an average value
σ_a	Uncertainty associated with a measurement, *a*
σ_{ER}	Experimental reproducibility; the error associated with replicate measurements of the same sample; reported in ‰
σ_{AE}	Acquisition error; the relative standard error associated with the ^{13}R measured across all scans
∂_y	partial derivative of a function, *y*
^{13}R	Ratio of ion counts of all singly substituted ^{13}C isotopologues for a given fragment vs the unsubstituted ^{12}C isotopologue
$^{13}R_{frag}$	^{13}R for a given mass fragment measured by the Orbitrap
$^{13}R_{sample,VPDB}$	^{13}R of a sample on the Vienna Pee Dee Belemnite scale, made possible where a standard has been measured using a complementary measurement approach on the VPDB scale
$^{13}R_{sample,Orbi}$	^{13}R of a sample as reported on the Orbitrap
$^{13}R_{standard,VPDB}$	13R of a standard on the Vienna Pee Dee Belemnite scale, measured via a complementary measurement approach.
$^{13}R_{standard,Orbi}$	13R of a standard as reported on the Orbitrap
Scan	A span of time (milliseconds) where the Orbitrap observes a packet of ions
VPDB	Vienna Pee Dee Belemnite; an international reference standard for carbon stable isotope analyses
Σ(x)	Sum of a given set of values, x
$\delta^{13}C_{Mol\,Avg}$	Molecular average $\delta^{13}C$ value for an analyte

Table S4. Molecular average and position-specific $\delta^{13}C_{VPDB}$ (‰) of purchased pyruvic acid standards, including the associated acquisition errors (σ_{AE}) and experimental errors (σ_{ER}).

		$\delta^{13}C$ (‰,VPDB)								
Sample	Method	MA (C1-3)	σ_{AE}	σ_{ER}	C1	σ_{AE}	σ_{ER}	C2+C3	σ_{AE}	σ_{ER}
Sigma A	GC-Orbitrap MS	-26.9	0.09	1.56	-30.5	0.16	2.83	-25.1	0.11	1.69
	GC-IRMS	-25.1	N/A	0.30	-30.2[a]	N/A	0.11	-22.5[a]	N/A	1.13
	GC py GC IRMS	-28.2	N/A	0.07	-29.9	N/A	0.08	-27.4	N/A	0.44
Sigma B	GC-Orbitrap MS	-23.5	0.11	0.69	-29.5	0.16	1.00	-20.5	0.14	0.57
	GC-IRMS	-27.6	N/A	0.30	N/A	N/A	N/A	N/A	N/A	N/A
	GC py GC IRMS	-27.3	N/A	0.13	-33.1	N/A	0.28	-24.4	N/A	0.20
Thermo B	GC-Orbitrap MS	-25.8	0.11	1.50	-30.1	0.20	2.08	-23.6	0.14	1.73
	GC-IRMS	-28.3	N/A	0.30	N/A	N/A	N/A	N/A	N/A	N/A
	GC py GC IRMS	-28.5	N/A	0.20	-33.4	N/A	0.48	-26.1	N/A	0.20
TCI	GC-Orbitrap MS	-8.5	0.07	1.04	-7.6	0.14	2.14	-8.9	0.08	1.25
	GC-IRMS	-7.8	N/A	0.38	N/A	N/A	N/A	N/A	N/A	N/A
	GC py GC IRMS	-7.3	N/A	0.16	-8.4	N/A	0.36	-6.8	N/A	0.24

[a] Intramolecular isotope signature of Sigma A measured on GC-IRMS following off-line sealed tube pyrolysis (Yamamoto & Back, 1984).

Table S5. Molecular average and position-specific $\delta^{13}C_{VPDB}$ (‰) of acetic acid standards, with acquisition errors (σ_{AE}) and experimental errors (σ_{ER}) reported for all methods.

		$\delta^{13}C$ (‰,VPDB)								
Sample	Method	MA (C1+C2)	σ_{AE}	σ_{ER}	C1	σ_{AE}	σ_{ER}	C2	σ_{AE}	σ_{ER}
Sigma	GC-Orbitrap MS	-19.1	0.03	0.05	-13.6	0.10	0.65	-24.6	0.12	0.70
	GC-IRMS	-18.6	N/A	0.23	N/A	N/A	N/A	N/A	N/A	N/A
Acros	GC-Orbitrap MS	-34.7	0.03	0.05	-34.0	0.08	2.76	-35.3	0.10	0.70
	GC-IRMS	-36.7	N/A	0.25	N/A	N/A	N/A	N/A	N/A	N/A
Millipore	GC-Orbitrap MS	-20.1	0.02	0.72	-20.4	0.07	1.28	-26.4	0.08	1.57
	GC-IRMS	-22.8	N/A	0.26	N/A	N/A	N/A	N/A	N/A	N/A
CRM	GC-Orbitrap MS	-27.1	0.02	0.89	-32.3	0.06	2.01	-21.8	0.08	2.21
	GC-IRMS	-29.2	N/A	0.22	N/A	N/A	N/A	N/A	N/A	N/A
AAA	GC-Orbitrap MS	-35.4	0.01	1.34	-26.9	0.04	1.63	-44.0	0.04	1.88
	GC-IRMS	-31.5	N/A	0.25	N/A	N/A	N/A	N/A	N/A	N/A
	Yamada et al. (2014)[a]	-32.3	N/A	0.12	-27.1	N/A	0.34	-37.5	N/A	0.32
AAB	GC-Orbitrap MS	-20.9	0.02	0.74	-15.4	0.05	1.09	-26.4	0.06	1.81
	GC-IRMS	-22.7	N/A	0.25	N/A	N/A	N/A	N/A	N/A	N/A
	Yamada et al. (2014)[a]	-22.3	N/A	0.02	-13.7	N/A	0.17	-31.0	N/A	0.17
AAD	GC-Orbitrap MS	-34.1	0.01	1.81	-23.9	0.04	1.50	-42.2	0.05	3.37
	GC-IRMS	-32.2	N/A	0.22	N/A	N/A	N/A	N/A	N/A	N/A
	Yamada et al. (2014)[a]	-35.9	N/A	0.02	-26.7	N/A	0.28	-45.0	N/A	0.28

[a]Samples noted above were analyzed at Tokyo Tech using the methods described in Yamada *et al* . (2014).

Analysis of Pyruvic Acid using the GC py-GC IRMS Method at Tokyo Tech.

Analysis of pyruvic acid standards were performed according to Nimmanwudipong et al.[2]. One mL of 100 mM aqueous pyruvate solution sample was added to a 10-mL gas-tight vial and sealed with a rubber cap for analysis. The pyruvate samples were then decarboxylated by adding 0.2 mL 30% hydrogen peroxide and allowed to react over 1 hour at room temperature. The samples were acidified following degradation by adding 50 µL phosphoric acid. The reaction mechanism (Scheme 1) can be seen below:

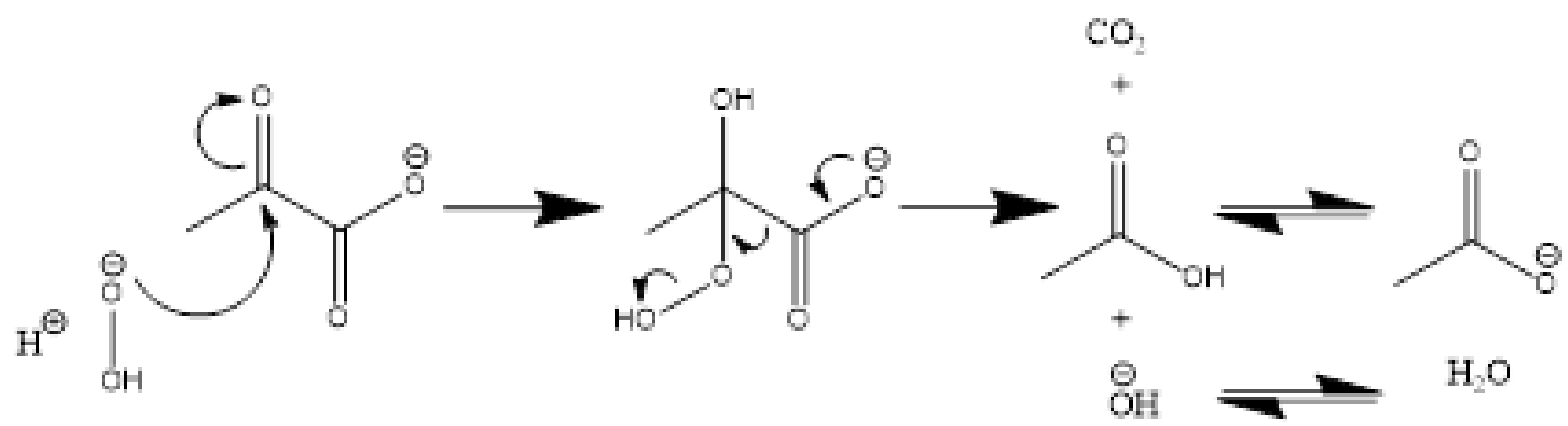

Scheme 1. Decarboxylation of pyruvate using hydrogen peroxide to acetic acid and CO_2.

The $\delta^{13}C$ value of evolved CO_2 (from the C_1 carboxyl carbon of pyruvic acid) was measured via gas chromatography (GC) coupled with an isotope ratio mass spectrometer (IRMS; Deltaplus XP, Thermo Fisher Scientific, Waltham, MA, USA) via a continuous-flow interface (GC Combustion III, Thermo Fisher Scientific, Waltham, MA, USA). The GC column used was a HP-PLOT-Q (30 m × 0.32 mm i.d., 10 mm film thickness; Varian, CA, USA) and the carrier gas was high purity helium at a flow rate of 1.5 mL/min. The run conditions of GC run were as follows: injector temperature 250℃; split mode; oven temperature was kept at 50℃. Isotopic standardization was made by CO_2 lab standards (-15.9±0.1‰ and -30.4±0.6‰).

The intramolecular $\delta^{13}C$ value of acetic acid, representing the C_2 ketone and C_3 methyl group from pyruvic acid, was measured using an on-line pyrolysis-GC-C-IRMS combined with head space solid-phase microextraction (HS-SPME)[3]. The system consists of a GC equipped with a capillary column (Nukol, 30 m × 0.32 mm i.d., 1 µm film thickness; Supelco, PA, USA), connected to a second GC equipped with a second capillary column (HP-Plot Q 30 m × 0.32 mm i.d., 20 µm film thickness; Agilent Technologies Inc., CA, USA). Two gas chromatographs were connected through a pyrolysis furnace (ceramic tube, 25 cm × 0.5 mm i.d.) operated at 1000°C for pyrolysis of acetic acid. The pyrolytic products were separated using the second capillary column and were introduced into a combustion furnace (ceramic tube, 25 cm × 0.5 mm i.d., packed with CuO, NiO, and Pt wires) operated at 960℃. The second GC was connected via a continuous flow interface to an IRMS.

A transfer line between chromatographs was made using deactivated fused silica capillary (0.32 mm i.d.; GL Sciences Inc., Japan). The acetic acid in the head space of the pyruvate degraded vials was collected on a SPME fiber (85 µm carboxen / polydimethylsiloxane (Carboxen/PDMS) StableFlex™; Supelco, Bellefonte, PA, USA) and injected into the GC-C-IRMS system. Collection of acetic acid on to the HS-SPME needle was conducted at 25℃ for 60 minutes. After HS-SPME extraction, the fiber was inserted into the injection port of the first gas chromatograph at 250℃. Helium was used as a carrier gas for all experiments. Chromatographic conditions were the following: 2.5 mL/min flow rate of carrier gas at a 2:1 split ratio. The first oven temperature program was the following: 50℃ (5 min), then rising to 200℃ (30 min) at the rate of 10℃/min. The second gas chromatograph was kept constant at 35℃. Isotopic standardization was accomplished by comparison with the reported sodium acetate reference materials (SAA and SAC)[3].

Analysis of Aqueous Acids using the 252 GC-IRMS, Including Sealed Tube Pyrolysis

Adapting the methods described in Thomas *et al.*[4], samples were acidified to a pH below the pKa of either pyruvic acid or acetic acid via the addition of oxalic acid. Oxalic acid was selected as the acidifying agent because it is non-volatile under the initial GC conditions and decomposes at temperatures >145ºC to CO_2, H_2O, and formic acid, thus reducing the potential degradation of the column media and metal catalysts in the combustion furnace. Using the same polar column used in the Orbitrap analyses (30 m x 0.25 mm x 0.25 µm thickness Supelco Nukol polar GC column with a 5 m x 0.25 mm deactivated guard column attached to the front end using a purged butt connector), aqueous samples were manually injected into a 220 ºC sample inlet with 1.4 mL/min He as the carrier gas. The GC column temperature was ramped as follows: 50 ºC for 1 minute, increased to 130 ºC at a rate of 10 ºC/min and held for 5 minutes, and then increased to 190ºC at a rate of 10ºC and held for 5 minutes for a total run time of 25 minutes. To avoid the introduction of water into the combustion furnace or the MS source, column flow was directed to vent for 400 seconds prior to switching the 4-port valve to direct the GC flow toward the 1020ºC Ni/Pt combustion furnace. The analytes then traveled through a Nafion water trap and an open split prior to being introduced to the MS[4].

Pyruvic acid subjected to sealed-tube pyrolysis was also analyzed via GC-IRMS as an additional complementary method to measure its position-specific isotope signature[5]. 20 uL of concentrated pyruvic acid was added to a quartz vessel, sealed under vacuum, and heated for at least 2 hours at 270ºC. Pyruvic acid thermally decarboxylates to produce CH_3CHO (comprised of the ketone and methyl carbons of pyruvic acid) and CO_2, where carbon dioxide is comprised entirely of the carboxyl (C_1) position of pyruvic acid. This CO_2 was measured via GC-IRMS equipped with a Rt-Q-BOND column (Restek; 30m x 0.25 mm x 8 µm). The sample inlet was set at 120ºC with 1.4 mL/min He as the carrier gas, and the oven temperature was kept constant at 30ºC for the entire run.

Table S6. Parameters used from representative acquisitions of pyruvic acid and acetic acid to calculate fragment-specific ionization efficiencies (E_{frag}) and shot noise limits (SNLs).

Acetic Acid (Acros)			Pyruvic Acid (TCI)		
**Fragment**	**43 m/z**	**45 m/z**	**Fragment**	**43 m/z**	**45 m/z**
**N_M (Ions)**	1.39E+10	3.03E+09	**N_M (Ions)**	1.04E+11	1.10E+10
**N_m (Ions)**	3.05E+08	2.91E+07	**N_m (Ions)**	2.51E+09	9.91E+07
**Scans**	4077		**Scans**	1424	
**^{13}R**	0.02239	0.00962	**^{13}R**	0.02408	0.00904
**Std. Error (^{13}R)**	1.54E-05	1.88E-05	**Std. Error (^{13}R)**	2.33E-05	4.09E-05
**Relative Std. Error (σ_{13R})**	6.89E-04	1.96E-03	**Relative Std. Error (σ_{13R})**	9.66E-04	4.53E-03
**Acquisition Error (σ_{AE})**	0.97	2.77	**Acquisition Error (σ_{AE})**	1.37	6.40
**SNL(σ_δ)**	0.08	0.26	**SNL(σ_δ)**	0.03	0.14
**E_{frag} (Ions/Molecule)**	2.22E-07	4.88E-08	**E_{frag} (Ions/Molecule)**	1.76E-06	1.82E-07
**σ_{AE} / SNL**	11.90	10.52	**σ_{AE} / SNL**	47.88	44.87

Supplementary References

(1) Bevington, P.; Robinson, K. *Data Reduction and Error Analysis for the Physical Sciences*, 3rd ed.; McGraw-Hill Education, 2002.

(2) Nimmanwudipong, T.; Gilbert, A.; Yamada, K.; Yoshida, N. Analytical Method for Simultaneous Determination of Bulk and Intramolecular ^{13}C-Isotope Compositions of Acetic Acid: Determination of Intramolecular Isotope Composition of Acetic Acid. *Rapid Commun. Mass Spectrom.* **2015**, *29* (24), 2337–2340. https://doi.org/10.1002/rcm.7398.

(3) Yamada, K.; Kikuchi, M.; Gilbert, A.; Yoshida, N.; Wasano, N.; Hattori, R.; Hirano, S. Evaluation of Commercially Available Reagents as a Reference Material for Intramolecular Carbon Isotopic Measurements of Acetic Acid: Reference Material for Intramolecular Carbon Isotopic Measurements. *Rapid Commun. Mass Spectrom.* **2014**, *28* (16), 1821–1828. https://doi.org/10.1002/rcm.6964.

(4) Thomas, B.; Freeman, K. H.; Arthur, M. A. Intramolecular Carbon Isotopic Analysis of Acetic Acid by Direct Injection of Aqueous Solution. *Org. Geochem.* **2009**, *40* (2), 195–200. https://doi.org/10.1016/j.orggeochem.2008.10.011.

(5) Yamamoto, S.; Back, R. A. The Photolysis and Thermal Decomposition of Pyruvic Acid in the Gas Phase. *Can. J. Chem.* **1985**, *63* (2), 549–554. https://doi.org/10.1139/v85-089.
